# Inflammatory myofibroblastic tumor of female genital tract with unusual features and potential diagnostic pitfalls

**DOI:** 10.3389/fonc.2025.1653181

**Published:** 2025-10-16

**Authors:** Huibin Zhang, Liyu Chen, Yuanqing Lin, Ting Lu, Yin Gao, Dan Luo, Shuxia Xu

**Affiliations:** ^1^ Department of Pathology, Fujian Maternity and Child Health Hospital, College of Clinical Medicine for Obstetrics & Gynecology and Pediatrics, Fujian Medical University, Fuzhou, Fujian, China; ^2^ Department of Pathology, Affiliated Hospital of Putian University, Putian, Fujian, China; ^3^ College of Environment and Public Health, Xiamen Huaxia University, Xiamen, Fujian, China

**Keywords:** inflammatory myofibroblastic tumor, female genital tract, ALK, epithelioid inflammatory myofibroblastic sarcoma, uterine mesenchymal tumor

## Abstract

**Background:**

Inflammatory myofibroblastic tumor (IMT) of the female genital tract is frequently misdiagnosed as uterine leiomyoma or mesenchymal stromal tumors due to overlapping morphological features.

**Materials and Methods:**

A total of 25 cases of IMT were collected between 2019 and 2024 and 8 of them were classified as IMT with unusual features. We conducted a retrospective analysis of 8 cases of unusual features IMT, focusing on histopathological features, immunophenotype, molecular alterations, and clinical follow-up.

**Results:**

The misdiagnosis rate is as high as 62.5%. Among these, 3 patients were initially misdiagnosed as uterine leiomyoma; 1 patient was misdiagnosed as low-grade endometrial stromal sarcoma; 1 patient was misdiagnosed as uterine leiomyosarcoma; and 3 patients remained unclear at initial assessment. The patient ranged from 32 to 67 (mean 43) years. Clinically, all presented with uterine masses. Tumors were either solitary or multiple, ranging from 1.2 to 12 cm. Histologically, these tumors exhibited marked heterogeneity with three predominant types, including leiomyoma-like type, myxoid type, and collagenous sclerosis type. 50% of the patients displayed a combination of two or more histologic subtypes. 2 of 8 (25%) patients presented leiomyoma-like morphology; 25% of patients exhibited prominent spiral arteriole proliferation resembling low-grade endometrial stromal sarcoma; 25% of patients occurring during pregnancy presented significant decidual-like changes; and 12.5% of patients resembled epithelioid leiomyosarcoma, characterized by frequent mitotic figures, severe nuclear atypia, prominent nucleoli, and abundant eosinophilic cytoplasm with epithelioid morphology. Immunohistochemical analysis revealed expression of ALK in 62.5% patients. The complete loss of p16 expression was noted in one patient, who was diagnosed as epithelioid inflammatory myofibroblastic sarcoma (EIMS). ALK gene rearrangements were identified by fluorescence in situ hybridization (FISH) on 62.5% patients. All tested cases were positive for ALK rearrangement. During clinical follow-up, 87.5% of patients followed a benign clinical course; the patient of EIMS developed pulmonary and supraclavicular lymph node metastases and remains living with tumor.

**Conclusions:**

EIMS has the ability of invasion and metastasis and presents the abnormal loss of p16. Accurate recognition of IMT with unusual features is crucial for targeted treatment. ALK protein expression and molecular testing play critical roles in diagnosis and differential diagnosis, and lowering the detection threshold to improve sensitivity is urgently needed.

## 1 Introduction

Inflammatory myofibroblastic tumor (IMT) is a spindle cell neoplasm, usually occurring in the soft tissues, lung, the abdominopelvic region, and viscera (1, 2). IMT of the female genital tract is a group of mesenchymal neoplasms with indeterminate biological behavior, which can progress from benign to malignant in clinical course (3). Due to overlapping morphological features with uterine stromal tumors, IMT is frequently misdiagnosed as uterine leiomyomas, smooth muscle tumors of uncertain malignant potential (STUMP), leiomyosarcomas, or even endometrial stromal sarcomas. Precise diagnosis is crucial because there are specific targeted treatment options available for IMT. Although recent advancements in pathologists’ recognition of IMT morphology and the application of anaplastic lymphoma kinase (ALK) immunohistochemistry and molecular testing have improved diagnostic accuracy, identifying atypical variants such as ALK-negative and uncommon histopathological characteristics of IMTs remain challenging. Herein, we retrospectively analyzed 8 cases of IMT of the female genital tract exhibiting rare and unusual pathological features, with the aim of summarizing their clinicopathological characteristics to improve diagnostic accuracy and reduce the risk of misdiagnosis.

## 2 Materials and methods

We define “ inflammatory myofibroblastic tumor with unusual features” as cases meeting any of the following criteria: (1) ALK immunohistochemistry-negative but confirmed by fluorescence *in situ* hybridization (FISH) to harbor ALK gene rearrangement; (2) histomorphology closely mimicking other uterine mesenchymal neoplasms (e.g., leiomyoma, endometrial stromal tumors), leading to a high likelihood of initial misdiagnosis; (3) belonging to rare histological subtypes (e.g., epithelioid inflammatory myofibroblastic sarcoma or decidual-like variant associated with pregnancy).

A total of 25 cases of IMT from Fujian Maternity and Child Health Hospital and Putian University Affiliated Hospital were collected between 2019 and 2024, and 8 of them were classified as IMT with unusual features. The age of the patients ranged from 32 to 67 years, with a median age of 41 years and an average age of 42.7 years. Clinical data were collected, including age, presenting symptoms, anatomical location, surgical procedure, and follow-up information.

All specimens were fixed in 10% neutral-buffered formalin and then embedded in paraffin, Afterwards, they were cut into sections with a thickness of 4 μm for hematoxylin and eosin (H&E) staining. Histomorphology evaluation was performed under light microscopy. Immunohistochemical staining was carried out using a fully automated immunostainer. The primary antibodies and chemical reagents were obtained from Maixin Biotech (Fuzhou, China), including ALK (clone D5F3), SMA, desmin, caldesmon, p16, p53, estrogen receptor (ER), progesterone receptor (PR), CD10, and Ki-67.

Fluorescence *in situ* hybridization (FISH) analysis for *ALK* gene rearrangement was performed using an *ALK* (2p23) break-apart probe kit (Anbiping Medical Technology, Guangzhou, China). A case was considered as positive when more than 15% of tumor cell presented the separation of red and green signals in the nuclei (i.e. separation diameter > sum of two signal diameters). For interpretation of immunohistochemical staining: focal positivity was defined as staining in 1-24% of tumor cells, moderate positivity as staining in 25-74% of tumor cells, diffuse positivity as staining in ≥75% of tumor cells, and negativity as no detectable staining.

## 3 Results

### 3.1 Clinical features

We analyzed 8 cases of IMT with unusual features. The clinical data were summarized in **Table 1**. The age of the patients ranged from 32 to 67 years, with a median age of 41 years and an average age of 42.7 years. Clinically, the main presenting symptoms included uterine mass and irregular vaginal bleeding. Among these, 3 cases were incidentally detected uterine masses during routine physical examination; 2 cases presented with uteroplacental masses during pregnancy; and the remaining 3 cases sought medical attention due to irregular vaginal bleeding. All patients were preoperatively diagnosed with “uterine space-occupying lesions” and underwent surgical resection. Surgical procedures included total hysterectomy with or without bilateral salpingo-oophorectomy in 4 cases, laparoscopic enucleation in 3 cases, and 1 case underwent dilatation and curettage based on endometrial assessment. Intraoperatively, tumors were observed as solitary or multifocal with size ranging from 1.2 to 12 cm, bearing variable demarcation from surrounding tissues (well-defined or infiltrative margins). Postoperatively, except for the patient with epithelioid inflammatory myofibroblastic sarcoma (EIMS), none of the other patients received any additional therapy.

**Table 1:** Clinical features of 8 cases of IMT with unusual features.

NO.	AGE	Chief complaint	Tumor location	Tumor size	Operative technique	Initial Diagnosis	Follow-up
1	32	A 38-week gestation with a coexisting uterine mass	Within the Placenta and Submucosa of the Uterus	3;1.2	Cesarean Section with Tumor Enucleation	Leiomyoma	NED, 60 mo
2	44	Uterine mass detected 2 years prior	Myometrium	3.5	THT+BS	Mesenchymal Tumors	NED, 39 mo
3	34	Uterine mass detected 11 months prior	Myometrium	8	Laparoscopic myomectomy	Leiomyoma	NED, 27 mo
4	47	Irregular vaginal bleeding for 2 weeks	Cervical internal os	3	Hysteroscopic endometrial curettage	Mesenchymal Tumors	NED, 16 mo
5	35	A 36-week gestation with a coexisting uterine mass	Submucosal	3.5	Laparoscopic myomectomy	Mesenchymal Tumors	NED, 15 mo
6	42	Irregular vaginal bleeding for 1 year	Submucosal	3	THT+BSO	Leiomyoma	NED, 17 mo
7	40	Uterine mass detected 3 months prior	Myometrium	5	THT	Low-grade endometrial stromal sarcoma	NED, 12 mo
8	67	Postmenopausal vaginal bleeding after 20 years of menopause	Myometrium	12	THT+BSO	Leiomyosarcoma	AWD, 14 mo

THT, Total Hysterectomy; BSO, Bilateral Salpingo-Oophorectomy; NED, no evidence of disease; AWD, alive with disease; mo, month.

### 3.2 Gross examination

Macroscopically, the 8 cases of IMT with unusual features resembled smooth muscle tumors in appearance, with most presenting as nodular masses. The tumor borders could not be clearly assessed in 4 cases due to surgical resection limitations; 3 cases exhibited relatively well-defined margins, and 1 case demonstrated infiltrative growth. The tumors were generally soft in consistency and yellowish in color, frequently showing areas of myxoid change, hemorrhage, and infarction.

### 3.3 Microscopic morphology

**Figure 1:**
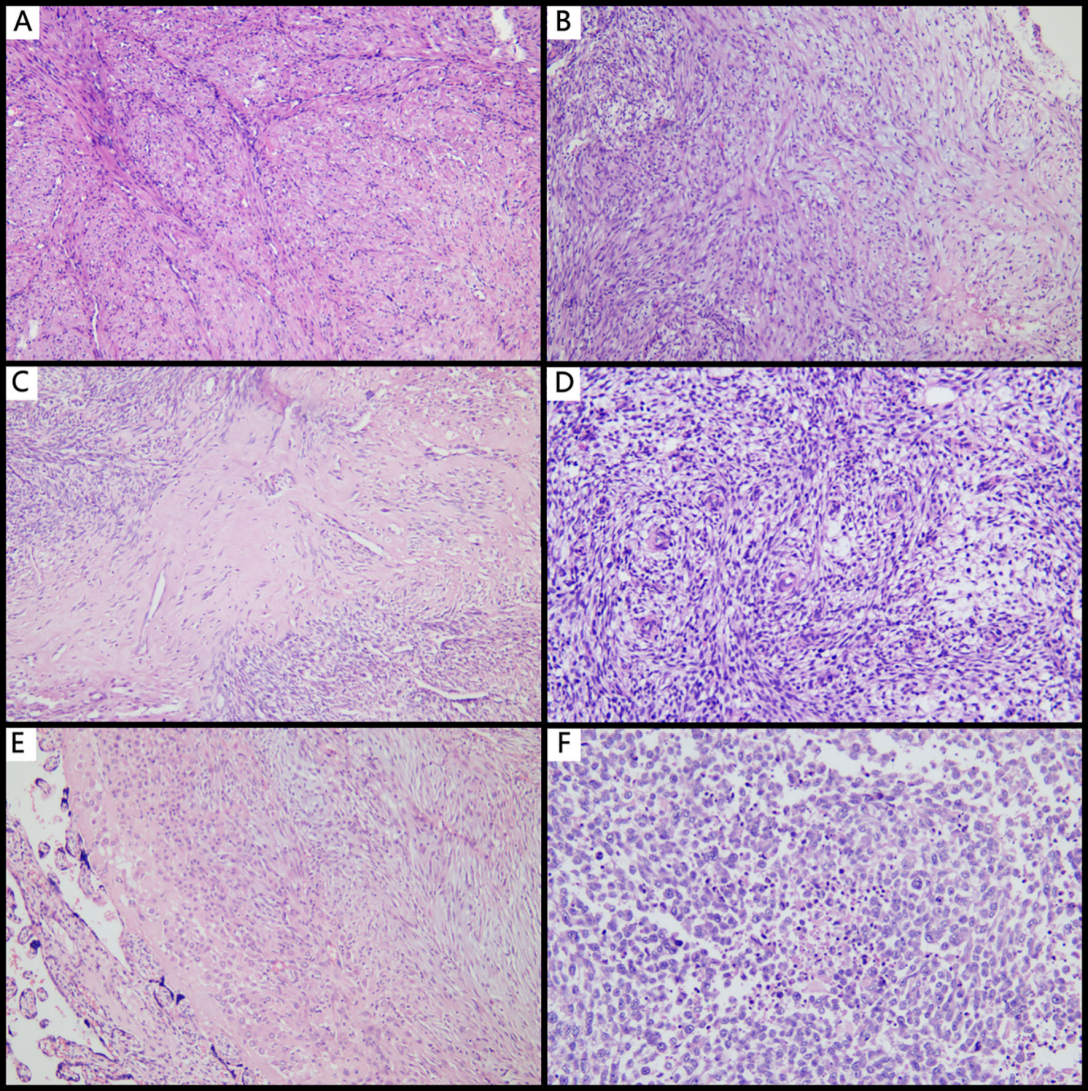
Histopathological features of IMT with unusual features. **(A)** Leiomyoma-like IMT mimicking leiomyoma (×100); **(B)** Myxoid type commonly seen in typical IMT (×100); **(C)** Collagenous sclerosing type demonstrating dense collagen deposition with sparse tumor cells (×100); **(D)** Tumor stroma rich in small spiral artery-like vessels (×100); **(E)** IMT within the placenta, showing decidual-like transformation (×100); **(F)** EIMS with extensive tumor necrosis and high-grade nuclear features (×100).

Microscopically, the tumors primarily displayed a mixed type, consisting of leiomyoma-like, myxoid, and collagenous morphologies, with only some cases presenting one or two of these types. Leiomyoma-like type was showed in **Figure 1A**: The tumor cells resembled smooth muscle tumor cells with eosinophilic cytoplasm and rod-shaped nuclei. Inflammatory infiltrate was sparse or absent in this area. Myxoid type was showed in **Figure 1B**: This type was characterized by loosely arranged myofibroblasts scattered within a background of myxoid stromal change, resembling nodular fasciitis. Collagenous type was showed in **Figure 1C**: This type was defined by hypocellular collagenous bands or acellular hyalinized zones with a few or no tumor cells present. The microscopic features of some cases were as follows: Cases 3 and 6 were initially diagnosed as leiomyomas and exhibited a purely leiomyoma-like histological pattern with no definitive assessment of tumor margins due to the surgical resection method. Case 1 occurred in the setting of pregnancy and was initially diagnosed as a pregnancy-associated leiomyoma. Case 7 was initially interpreted as a low-grade stromal sarcoma. Both cases showed prominent stromal vascular proliferation (**Figure 1D**) with tumor cells displaying short spindle to decidual-like morphology (**Figure 1E**). Case 8 (EIMS) was initially diagnosed as leiomyosarcoma.

### 3.4 Immunophenotype

**Figure 2:**
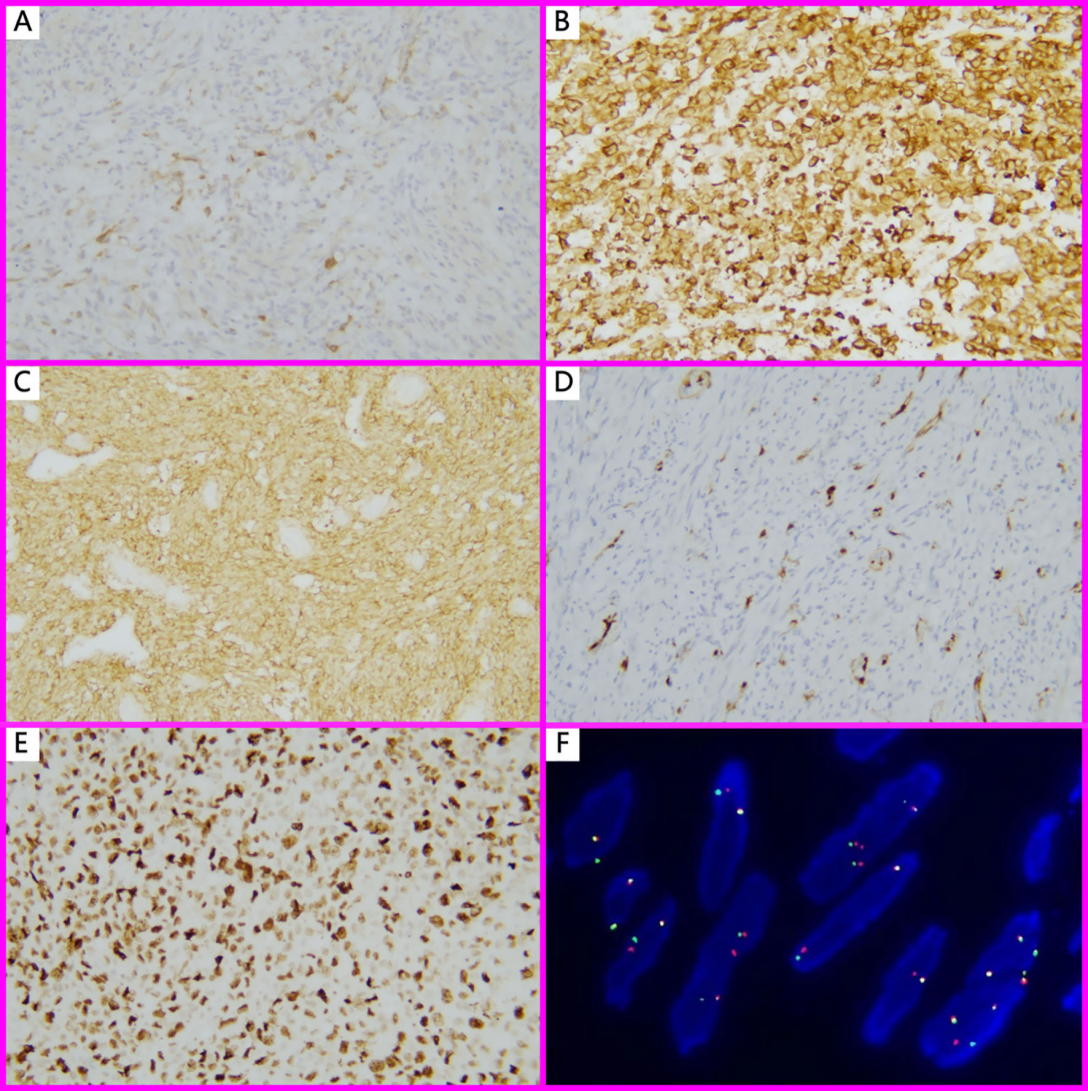
Immunohistochemistry and FISH findings in IMT with unusual features. **(A)** ALK 1+, Focal positivity (Immunohistochemistry, ×100); **(B)** ALK3+, in EIMS case (Immunohistochemistry, ×200); **(C)** CD10 strongly positive in a spiral-artery-rich variant of IMT (Immunohistochemistry, ×200); **(D)** Except for EIMS, all other IMT with unusual features showed focal or mosaic p16 staining (Immunohistochemistry, ×100); **(E)** EIMS showed a high Ki-67 proliferation index (90%) (Immunohistochemistry, ×200); **(F)**. FISH analysis revealed *ALK* (2p23) gene rearrangement, characterized by split red and green signals in more than 15% of tumor nuclei.

Most cases (62.5%) showed positivity for ALK, with staining localized to the cytoplasm and varying from focal to diffuse in distribution (**Figures 2A, B**). Desmin was expressed in 6 of 7 cases. CD10, ER, and PR were variably expressed in 5 of 6 cases (**Figure 2C**). 2 of 6 cases presented the expression of h-Caldesmon. Focal to moderate mosaic expression of p16 was observed in 4 of 5 cases (**Figure 2D**), while 1 case showed complete loss of p16 expression, indicating the abnormal pattern. All tested cases (5/5, 100%) showed wild-type expression of p53. The Ki-67 proliferation index ranged from 3% to 90% (**Figure 2E**). Detailed immunohistochemical and pathological features were summarized in **Table 2**.

**Table 2:** Pathological features of 8 cases of IMT with unusual features.

Case NO.	Histological features			Immunophenotype									FISH
	L-T	M-T	CS-T	ALK	Desmin	H-Cadelsmon	CD10	ER	PR	p16	p53	Ki-67	*ALK* (2p23)
1	–	+	–	3+	UK	UK	UK	UK	UK	UK	UK	UK	UK
2	+	+	–	0	2+	2+	0	1+	3+	2+	WT	5%+	+
3	+	–	–	2+	2+	2+	2+	1+	3+	UK	WT	3%+	UK
4	+	+	–	0	0	0	2+	2+	3+	1+	WT	3%+	+
5	–	+	+	0	2+	0	2+	2+	3+	1+	WT	5%	+
6	+	–	–	1+	3+	UK	UK	UK	UK	UK	UK	3%	+
7	–	+	–	2+	3+	0	3+	2+	3+	2+	WT	30%	UK
8	+	+	–	3+	1+	0	2+	0	0	0	WT	90%	+

L-T, Leiomyoma-like type; M-T Myxoid type; CS-T, Collagenous sclerosing type; -, Absent; +, Present; 0, negative; 1+, weak positivity; 2+, moderate positivity; 3+, strong positivity; UK, Unknown; WT, Wild Type.

### 3.5 FISH analysis

FISH analysis for *ALK* gene rearrangement at locus 2p23were performed on 62.5% cases of IMT with unusual features. All tested cases (5/5, 100%) showed *ALK* gene rearrangement (**Figure 2F**), including 3 cases that were negative for ALK protein expression by immunohistochemistry, 1 case exhibiting a leiomyoma-like histological pattern, and 1 case of EIMS.

### 3.6 EIMS case

The EIMS case presents aggressive behavior and unique morphology. It presented with a massive tumor, measuring up to 12 cm in diameter. The cut surface revealed that tumors presented extensive myxoid degeneration, ill-defined borders, and infiltrative invasion into the muscular layer with focal transmural extension through the serosal layer, suggesting its locally aggressive behavior. It also presented a unique morphology. Microscopically, the tumor cells exhibited abundant eosinophilic cytoplasm and severe nuclear atypia, ranging from plump spindle to polygonal shapes (**Figure 1F**). Notable features included prominent large nucleoli, brisk mitotic activity (>10 mitoses per 10 high-power fields), and areas of tumor necrosis (**Figure 2E**). Moreover, numerous intravascular tumor emboli were identified. Another unique feature of EIMS is the complete loss of p16 expression.

### 3.7 Follow-up

Clinical follow-up ranged from 12 to 60 months. Among the patients, Case 8(EIMS) developed pulmonary and left supraclavicular lymph node metastases after surgery for 8 months and has been living with tumor for 14 months after recurrence. Other patients remained tumor-free during the follow-up period.

## 4 Discussion

IMT of the female genital tract is relatively rare and can occur in various anatomical sites, including the cervix, uterine corpus, adnexa, parametrial soft tissues, pelvis, and placenta. The most common locations are the submucosal and intramural regions of the uterine corpus. Currently, the recurrence rate and metastasis rate of IMT is about 25% and 2%, respectively (4). Patients may present at a wide age range from 3.5 to 78 years (4–6). Clinically, these tumors typically manifest as “uterine masses”, either asymptomatic or accompanied by symptoms related to mass effect, such as vaginal bleeding, abdominal distension, and pelvic pain. These clinical features are essentially the same as those observed in the IMT with unusual features described in our series, highlighting that it is often not feasible to differentiate between typical IMT and cases exhibiting unusual features based on clinical features alone.

IMT of female genital tract with unusual features is often misdiagnosed. We incidentally identified and corrected two cases (Case 3 and Case 6), which were initially misdiagnosed as leiomyomas and subsequently reclassified as IMT of the uterus. Inspired by this finding, a retrospective review of previously diagnosed or suspected cases of IMT in the pathology files was performed, which ultimately led to the identification of 8 cases of IMT with uncommon morphological features. These atypical variants of IMT were rarely reported in the literature and were associated with an ultrahigh rate of misdiagnosis (62.5%). By systematically analyzing their histopathological characteristics and comparing them with those of classical IMT, we aim to identify key diagnostic clues and distinguishing features. Interestingly, during gross examination and tissue sampling, we identified several specific and useful morphological features of IMT with unusual features: the presence of myxoid change, heterogeneous consistency with focally softer areas, focal yellowish discoloration, and ill-defined tumor margins.

The histological morphology of IMT of the female genital tract is highly variable. Rabban et al. (7) summarized three major histologic patterns: a hypocellular pattern, a fascicular pattern, and a hyalinized pattern. The definition of IMT by Rabban et al. is also adopted by the WHO. Nowadays, the histological patterns are often referred to as: myxoid, hypercellular, and fibrous hypocellular. We observed that the microscopic features of IMT included three histological patterns: myxoid type, leiomyoma-like type, and collagenous sclerosing type. The myxoid type resembled nodular fasciitis, characterized by a loose proliferation of myofibroblast-like tumor cells embedded in a myxoid stroma, often accompanied by inflammatory cell infiltration and prominent vascularity. The leiomyoma-like type resembled uterine leiomyomas, with spindle-shaped tumor cells exhibiting elongated, plump nuclei arranged in fascicles or a storiform pattern. The collagenous sclerosing type was defined by sparse tumor cells embedded within dense collagenous stroma, showing hyalinization-like changes. Typical IMT mainly exhibits a mixture of the aforementioned histological patterns, although they may also present as a single predominant pattern. In all cases, a variable inflammatory infiltrate was present in the background, ranging from sparse to diffuse, and predominantly composed of lymphocytes and plasma cells. Recently, a rare variant with prominent inflammatory features, referred to as the “hyperinflammatory subtype”, has also been reported (8). The tumor cells in IMT typically show scant to moderate eosinophilic cytoplasm and spindle-shaped nuclei that usually exhibits mild to moderate atypia and inconspicuous nucleoli. Among rare instances, the tumor cells may display an epithelioid morphology, characterized by vesicular nuclei and prominent nucleoli, which is a feature characteristic of EIMS (9). Moreover, for the morphological changes of placental or uterine IMT occurring during pregnancy, in addition to the typical histological features of IMT, decidual-like transformation of the tumor cells can also be observed (10). Nicholas et al. (10) confirmed that the placenta-associated IMT originated from uterine through short tandem repeat (STR) genotyping. Among the 8 cases of IMT with unusual features reported here, 2 cases occurred during pregnancy. Notably, one of these cases presented not only a well-circumscribed mass within the uterine cavity, but also a distinct lesion within the placental parenchyma that was contiguous with the basal decidua. This finding suggests that placental IMT and uterine IMT may share the same origin and IMT may extend from the uterus into the placenta.

None of the 8 cases of IMT with unusual features exhibited histological features of the hyperinflammatory subtype. 2 cases displayed a purely leiomyoma-like morphology and were both initially misdiagnosed as leiomyomas. Another 2 cases showed prominent stromal vascularity, where one was misdiagnosed as a pregnancy-associated leiomyoma and the other was misdiagnosed as a low-grade endometrial stromal sarcoma (diffusely CD10-positive). Although ALK-negative, because containing classic histological patterns of IMT, such as myxoid and collagenous areas, the remaining 4 cases were therefore not misdiagnosed. Moreover, in pregnancy-associated cases, the presence of myxoid change and decidual-like transformation may predispose pathologists to the diagnosis of degenerative leiomyomas, thereby resulting in the insufficient recognition of these lesions.

The specificity and sensitivity of ALK immunohistochemistry in the diagnosis of IMT have been well established. In general, ALK positivity is reported in over 80% of uterine IMT. However, IMT may also present variable expression of other markers, including ER, PR, desmin, SMA, h-caldesmon, and CD10 (11, 12), which can complicate the differential diagnosis. This is why the two patients were misdiagnosed in our case series. p53 always seems to be wild-type expressed, whereas p16 does not seem to be helpful for diagnosis. Nevertheless, it has been reported that abnormal loss or overexpression of p16 are associated with more aggressive biological behavior and poorer clinical outcomes (13). Herein, in the 8 cases of IMT with unusual features, the ALK positivity rate was 62.5%. All 5 cases showed wild-type expression of p53. Regarding to p16, focal to moderate staining was observed in all cases, except for the EIMS case exhibiting the complete loss of p16. These results support the hypothesis that aberrant p16 expression may be associated with malignant biological behavior in IMT.

FISH is the gold standard for detecting *ALK* gene rearrangements, which are present in more than 75% of IMT of the female genital tract. A variety of fusion genes have been reported in *ALK*-rearranged IMT of the female genital tract, including *IGFBP5, THBS1, FN1, TIMP3, TPM3, TPM4, EML4, CTCL, RANBP2, SEC31A, DES, and DCTN1* (14). Accompanying gene rearrangements of *ROS1*, *NTRK1/3*, *RET*, and *PDGFRβ* or accompanying gene fusions of *ETV6-NTRK3*, *TIMP3-ROS1*, and *TIMP3-RET* were detected in a few of non-*ALK*-rearranged IMT (14–17). Among the 8 cases of IMT with unusual features in our series, FISH analysis was performed on 5 cases, including 3 cases of ALK-negative IMT, 1 case of pure leiomyoma-like IMT, and 1 case of EIMS. Interestingly, all of them were found to harbor *ALK* gene rearrangements. These findings reveal the critical role of FISH testing in confirming the diagnosis of IMT, particularly in morphologically atypical cases. Although harboring *ALK* gene rearrangement, 3 cases still present ALK-negative. This may be owing to the ALK expression below the detection threshold. Therefore, lowering the detection threshold will be beneficial in reducing misdiagnoses.

Because the IMT of the female genital tract with unusual features is frequently misdiagnosed as uterine mesenchymal neoplasms. Therefore, multiple identification methods should be taken. The integration of immunohistochemical and molecular testing can significantly help to distinguish these two types of tumors. In general, uterine tumors that are positive for ALK protein or harbor *ALK* gene rearrangements should preferentially raise suspicion for IMT. In differential diagnosis, IMT with unusual features should be primarily distinguished from uterine smooth muscle tumors. IMT with a purely leiomyoma-like morphology and bland nuclei may be mistaken for benign leiomyomas, while those showing mild to moderate nuclear atypia may resemble smooth muscle tumor of uncertain malignant potential (STUMP) (14). The epithelioid variant EIMS may be confused with leiomyosarcoma. However, several features may facilitate the diagnosis of IMT: the presence of an inflammatory background is commonly observed; most cases show ALK expression on immunohistochemistry; and there is often reduced or absent staining for desmin, h-caldesmon, ER, and p16. Molecular testing typically presents *ALK* gene rearrangement. In contrast, STUMP almost not express ALK and constantly express two or three myogenic markers. Moreover, IMT with unusual features must be differentiated from endometrial stromal tumors (ESTs). CD10 is often regarded as a useful marker for ESTs, but it is not entirely specific. Studies have shown that a considerable proportion of IMT also express CD10 (18). When accompanied by prominent vascular proliferation, IMT may be very similar to ESTs histologically, leading to the misdiagnosis. Fortunately, neither low-grade nor high-grade ESTs typically express ALK (18), and they were characterized by distinct molecular genetic alterations. For instance, high-grade ESTs frequently harbor *YWHAE-NUTM2A/B* or *ZC3H7B-BCOR* fusions. Therefore, it is feasible to distinguish IMT with unusual features and ESTs through the analysis of ALK expression. Finally, IMT with unusual features should also be distinguished from other rare mesenchymal neoplasms such as solitary fibrous tumor (SFT) and fibromatosis. SFTs are consistently ALK-negative and typically express STAT6, CD34, BCL-2, and CD99. Similarly, fibromatosis also lacks ALK expression and is commonly associated with abnormal nuclear accumulation of β-catenin due to mutations in the *CTNNB1* gene.

IMT has the potential of local recurrence and metastasis. Several clinicopathological features of IMT have been associated with poor prognosis, including age over 45 years, size of tumor greater than 5 cm, tumor necrosis, mitotic count ≥4 per 10 high-power fields (HPF), nuclear atypia, infiltrative margins, and vascular invasion (18, 19). Recurrence has been reported in cases of purely leiomyoma-like IMT (8). Currently, there is no generally accepted treatment protocol for IMT. Surgical excision, particularly the total hysterectomy, remains the preferred treatment. Notably, patients harboring *ALK* gene rearrangements may benefit from tyrosine kinase inhibitors (TKIs), as these tumors may be sensitive to targeted therapy with TKIs (20, 21). Crizotinib, a small-molecule TKI targeting ALK, has shown strong clinical activity and successfully applied to non-small-cell lung cancer therapy with *ALK* rearrangements (21). This TKI also has been reported to be effective in the treatment of patients with IMT (21). Among the 8 patients of IMT with unusual features, 4 patients underwent total hysterectomy, 3 patients underwent laparoscopic enucleation, and 1 patient had endometrial curettage. Clinical follow-up ranged from 12 to 60 months. Excitingly, 87.5% of patients had a benign clinical course and now remain tumor-free. However, the remaining patient with EIMS developed lung and left supraclavicular lymph node metastases after surgery for 8 months and is currently living with the tumors for 14 months after tumor recurrence. These results demonstrate EIMS has the ability of invasion and metastasis. Early intervention should be taken on it.

## 5 Conclusion

In summary, IMT of the female genital tract with unusual features is a rare tumor with ultrahigh misdiagnosis rate. Recognizing this tumor can be challenging on routine hematoxylin and eosin (H&E) staining, especially in the absence of a prominent inflammatory background or when tumor cells display only a monotonous leiomyoma-like morphology, or are associated with a stroma rich in spiral arterioles. When a tumor in female genital tract present with soft in consistency, yellowish in color, and variable demarcation from surrounding tissues, IMT with unusual features should be considered. In these scenarios, immunohistochemical detection of ALK protein expression, together with molecular genetic analysis of *ALK* gene rearrangement by FISH, can provide valuable evidences for establishing an accurate diagnosis. Moreover, identifying *ALK* gene rearrangements not only aids in differential diagnosis, but also may open up the possibility for targeted therapy with TKIs, offering great benefits to patients.

## Data Availability

The raw data supporting the conclusions of this article will be made available by the authors, without undue reservation.
